# Diversity of the CD4 T Cell Alloresponse: The Short and the Long of It

**DOI:** 10.1016/j.celrep.2015.12.099

**Published:** 2016-01-21

**Authors:** Jason M. Ali, Margaret C. Negus, Thomas M. Conlon, Ines G. Harper, M. Saeed Qureshi, Reza Motallebzadeh, Richard Willis, Kourosh Saeb-Parsy, Eleanor M. Bolton, J. Andrew Bradley, Gavin J. Pettigrew

**Affiliations:** 1 University of Cambridge, School of Clinical Medicine, Cambridge CB2 0QQ, UK; 2 NIH Tetramer Facility, Emory/Yerkes, 954 Gatewood Road, Atlanta, GA 30329, USA

## Abstract

MHC alloantigen is recognized by two pathways: “directly,” intact on donor cells, or “indirectly,” as self-restricted allopeptide. The duration of each pathway, and its relative contribution to allograft vasculopathy, remain unclear. Using a murine model of chronic allograft rejection, we report that direct-pathway CD4 T cell alloresponses, as well as indirect-pathway responses against MHC class II alloantigen, are curtailed by rapid elimination of donor hematopoietic antigen-presenting cells. In contrast, persistent presentation of epitope resulted in continual division and less-profound contraction of the class I allopeptide-specific CD4 T cell population, with approximately 10,000-fold more cells persisting than following acute allograft rejection. This expanded population nevertheless displayed sub-optimal anamnestic responses and was unable to provide co-stimulation-independent help for generating alloantibody. Indirect-pathway CD4 T cell responses are heterogeneous. Appreciation that responses against particular alloantigens dominate at late time points will likely inform development of strategies aimed at improving transplant outcomes.

## Highlights

• Direct-pathway CD4 T cell alloresponses are extremely short lived
• Indirect pathway responses vary markedly according to target alloantigen
• In chronic rejection, MHC class I alloantigen is continually processed and presented
• The kinetics of the alloreactive CD4 T cell response are altered profoundly

## Graphical Abstract

**Figure:**
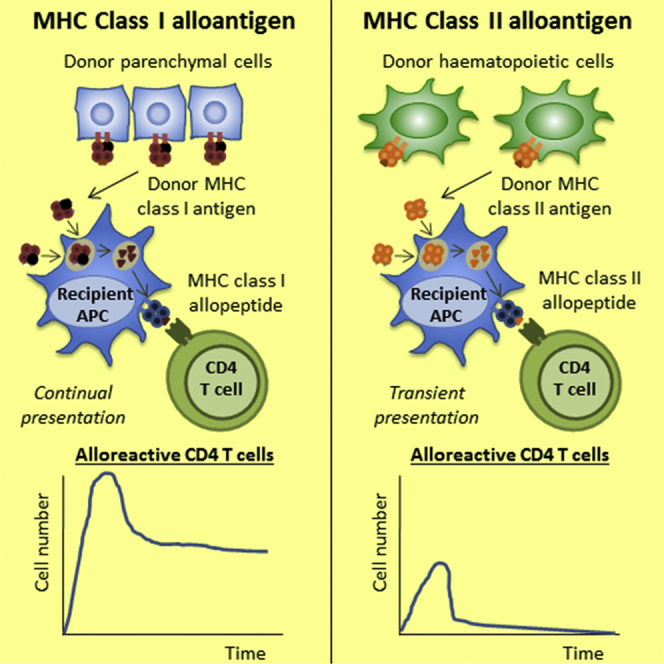

## Teaser

The T cell allorecognition pathways governing rejection of organ allografts are poorly understood. Using a model of chronic heart graft rejection, Ali et al. show that continual presentation of MHC class I allopeptide drives late division in the responding alloreactive CD4 T cell population, resulting in a markedly augmented maintenance phase.

## Introduction

Chronic rejection, leading to late graft loss, remains the major challenge for solid organ transplantation. T cells play a critical role in the development of chronic rejection (Ali et al., 2013, Libby and Pober, 2001), but it is not clear whether the early T cell response following transplantation is sufficient to mediate chronic rejection or, as seems more likely, persistent alloantigen-driven T cell responses are required over a longer period of time.

CD4 T cells recognize alloantigen through two distinct pathways. In the “direct pathway,” alloreactive T cells recognize intact donor MHC molecules presented on the surface of donor antigen-presenting cells (APCs), whereas in the “indirect pathway,” T cells recognize major, and minor, histocompatibility antigens that have been acquired by recipient APCs, processed and presented as self-MHC-restricted peptides (Ali et al., 2013, Jiang et al., 2004). The relative contribution of these pathways to chronic graft rejection remains unclear (Benichou, 1999, Gould and Auchincloss, 1999, Nadazdin et al., 2011). It has generally been assumed that direct-pathway CD4 T cell alloresponses are short lived due to rapid destruction of donor APCs following transplantation. Consequently, chronic rejection is considered to be largely mediated by indirect-pathway CD4 T cell responses (Baker et al., 2001, Ciubotariu et al., 1998, Haynes et al., 2012, Hornick et al., 2000, Safinia et al., 2010). However, late direct-pathway responses have been reported in primate studies (Nadazdin et al., 2011), possibly reflecting upregulated expression of MHC class II on allograft endothelium. Similarly, the indirect CD4 T cell allorecognition pathway is generally regarded as a single entity but is instead presumably a culmination of multiple responses against potentially every disparate alloantigen expressed by the graft. Given that these antigens are likely to be expressed at different concentrations in the graft and, in the case of MHC class II, predominantly expressed on the hematopoietic components of the graft, it is plausible that the duration and strength of indirect-pathway responses differ depending on the target alloantigen. This concept has yet to be examined definitively.

Here, we show in a murine model of chronic allograft rejection that direct-pathway CD4 T cell responses are short lived but also that indirect-pathway responses are heterogeneous and vary markedly according to target antigen. Whereas those directed against MHC II allopeptide decline rapidly after transplant, the persistent presentation of immunogenic target epitope provokes continued division of MHC class I allopeptide-specific CD4 T cells and results in a markedly augmented late maintenance phase. Anamnestic function in this expanded population is nevertheless sub-optimal. The implications of our findings to late graft rejection are discussed.

## Results

### Experimental Approach and Characterization of Transplant Model

**Figure 1:**
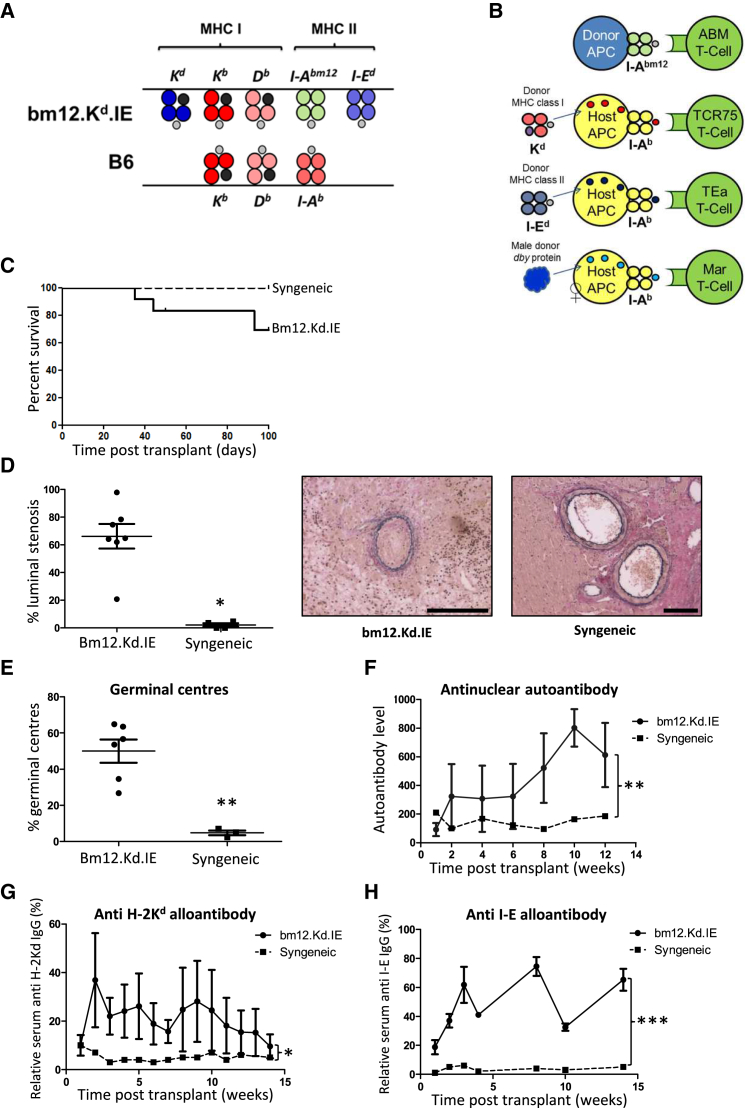
Characterization of MHC-Mismatched Murine Model of Chronic Heart Allograft Vasculopathy (A) B6.Kd, bm12, and ABOIE mice were inter-crossed to create a “bm12.kd.IE” donor strain that differed from the C57BL/6 recipient strain at the classical MHC class I H-2K and MHC class II I-A and I-E loci, enabling direct-pathway CD4 T cell alloimmunity to be assessed by adoptive transfer of CFSE-labeled, TCR-transgenic ABM CD4 T cells. (B) I-A^b^-restricted indirect pathway CD4 T cell responses against MHC class I H-2K^d^, MHC class II IE, and minor H-Y *dby* antigen could be similarly assessed by transfer of TCR-transgenic TCR75, Tea, and Marilyn CD4 T cells, respectively. (C and D) Whereas syngeneic C57BL/6 heart grafts survive indefinitely, bm12.kd.IE heart allografts are rejected slowly by C57BL/6 recipients (C) with development of progressive allograft vasculopathy (D): representative photomicrographs of elastin van-Gieson-stained paraffin sections depicting typical fibroproliferative arterial intimal thickening observed in rejecting heart allografts (scale bars, 100 μm). (E–H) Rejection is associated with development of germinal centers (E), class-switched anti-nuclear autoantibody (F), and anti H-2K^d^ (G) and anti-I-E (H) alloantibody responses. ^∗^p < 0.05, ^∗∗^p < 0.01, and ^∗∗∗^p < 0.001 (Kaplan-Meier log rank analysis in C, Mann-Whitney test in D and E, and two-way ANOVA in F–H). Data represent mean and SEM of n = 7 mice per group.

To examine the CD4 T cell allorecognition pathways active at early and late time points after transplantation, a donor strain (bm12.Kd.IE) was created that differed from the C57BL/6 recipient strain at the I-A^bm12^ and I-E^d^ MHC class II and H-2K^d^ MHC class I loci (Figure 1A), enabling direct and indirect CD4 T cell recipient alloresponses to be assessed by adoptive transfer of populations of TCR-transgenic CD4 T cells with precise specificity for alloantigen. Following transplantation of male bm12.Kd.IE hearts into female C57BL/6 recipients, direct-pathway CD4 T cell responses against MHC class II I-A^bm12^ alloantigen were assessed by quantifying division of adoptively transferred ABM CD4 T cells. Indirect-pathway CD4 T cell responses against I-A^b^-restricted MHC class I H-2K^d^ alloantigen, MHC class II I-E alloantigen, and minor male H-Y alloantigen were assessed by division of adoptively transferred TCR75, TEa, and Marilyn CD4 T cells, respectively (Figure 1B): these T cell clones do not recognize donor I-A^bm12^-restricted alloantigen (Figure S1). Bm12.Kd.IE heart allografts were not rejected acutely (Figure 1C) but showed progressive allograft vasculopathy (Figure 1D), with rejection characterized by development of germinal center (GC) anti-class I (H-2K^d^) and anti-class II (I-E) alloantibody and anti-nuclear autoantibody responses (Figures 1E–1H).

### Heterogeneity of Indirect-Pathway CD4 T Cell Responses

**Figure 2:**
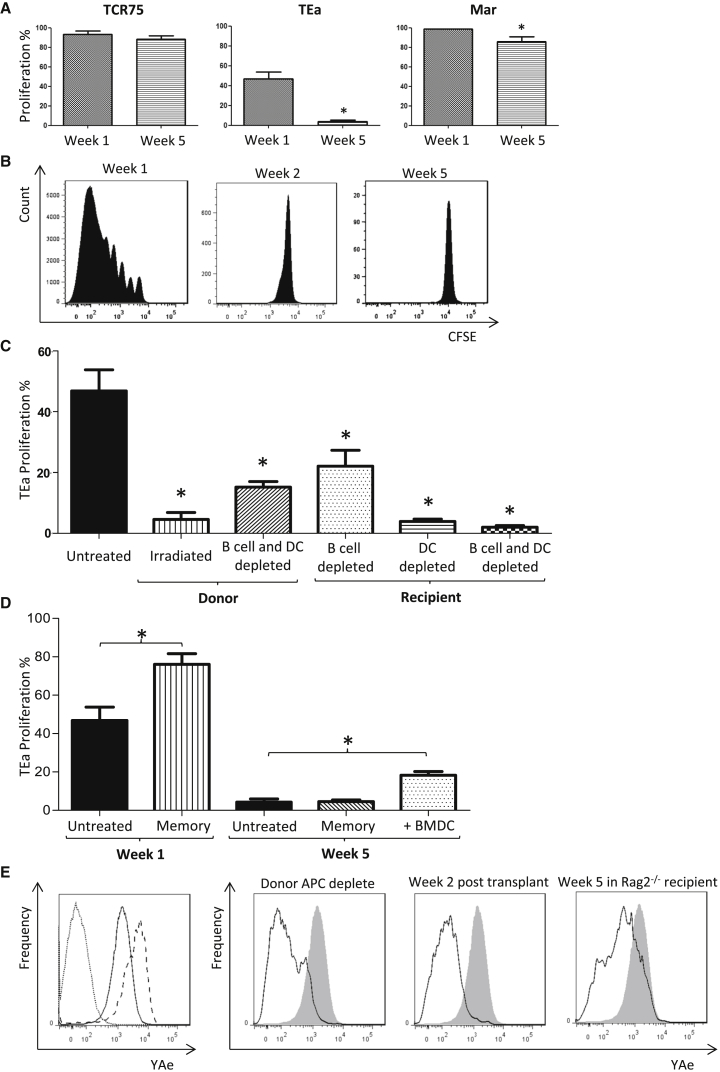
Heterogeneity of Indirect-Pathway CD4 T Cell Responses (A) Following transplantation of female C57BL/6 recipients with a male bm12.Kd.IE heart allograft, indirect pathway CD4 T cell responses against mismatched MHC class I H-2K^d^, MHC class II I-E, and minor H-Y antigen were assessed by quantifying division (expressed as percentage of cells undergoing at least one division) of CFSE-labeled TCR75, Tea, and Marilyn CD4 T cells, respectively, transferred at either transplantation or 5 weeks later. (B) Whereas CFSE-labeled TEa CD4 T cells proliferate robustly when transferred at transplantation of a C57BL/6 recipient with a bm12.Kd.IE heart allograft, no division is observed when TEa CD4 T cells are transferred 1 week later. (C) Division profiles of TEa CD4 T cells transferred to C57BL/6 recipients at transplantation with bm12.Kd.IE heart allografts, with the donors lethally irradiated or either the donor or recipient mice depleted of B cells and/or DCs. Division profiles following transfer of memory TEa CD4 T cells to C57BL/6 recipients of a bm12.Kd.IE heart allograft are shown. Profiles of transferred naive TEa CD4 T cells are replicated from (A) for comparison. (D) Also shown is division of naive TEa CD4 T cells transferred 5 weeks after transplantation to a C57BL/6 recipient that received bm12.Kd.IE BMDCs the day before TEa transfer. Statistical comparison is to untreated group. (E) YAe clonotypic antibody was used to identify I-A^b^-restricted I-E peptide epitope on purified DCs. (Left panel) Overlay histogram of YAe staining of splenic DCs from naive C57BL/6 mice (dotted), naive BALB/c x C57BL/6 F1 (CB6F1) mice (dashed), and DCs purified from C57BL/6 recipients 5 days after bm12.Kd.IE heart transplantation (solid) is shown. (Right panel) Comparative YAe staining of splenic DCs from C57BL/6 recipients 1 week after transplantation with DC- and B cell-depleted bm12.Kd.IE heart allografts; C57BL/6 recipients 2 weeks after bm12.Kd.IE heart transplantation; and H-2^b^*Rag2*^*−/−*^ recipients 5 weeks after bm12.Kd.IE heart transplantation is shown (DCs purified from C57BL/6 recipients 5 days after bm12.Kd.IE heart transplantation are overlaid for comparison—shaded). ^∗^p < 0.05, ^∗∗^p < 0.01, and ^∗∗∗^p < 0.001 (Mann-Whitney test in A, C, and D). Data are representative of two independent experiments resulting in n = 5 in each group (A, C, and D; mean and SEM of n = 5 mice per group).

Responses of the transferred allospecific CD4 T cell clones to the heart allograft were quantified by carboxyfluorescein succinimidyl ester (CFSE) division profiles (Figure S2) but with the modification that CD4 T cell clones were transferred early, at transplantation, or late on day 28 after transplant (to measure CD4 T cell alloresponses during the first and fifth week, respectively). We reasoned that division of the transferred CD4 T cells would be contingent upon expression of, and stimulation by, the appropriate alloantigen epitope (Obst et al., 2005). In the first week after heart transplantation, robust indirect-pathway CD4 T cell responses were detected against H-2K^d^ MHC class I, I-E MHC class II, and H-Y alloantigen (Figure 2A). At 5 weeks, the responses against MHC class I allopeptide and H-Y antigen were preserved, albeit the anti-H-Y response was slightly attenuated. In contrast, the anti-I-E response was undetectable (Figure 2A).

### Indirect Alloresponses against MHC Class II Alloantigen Are Limited by Antigen Availability

Further experiments revealed that the anti-I-E indirect-pathway response decayed by week 2 (Figure 2B). To test the hypothesis that this reflected elimination of donor hematopoietic cells (the likely major source of MHC class II alloantigen), anti-MHC class II indirect-pathway responses were evaluated following transplantation with bm12.kd.IE hearts from donors that had either been subject to lethal irradiation or depleted of the dendritic cell (DC) fraction. Both approaches resulted in marked attenuation of the early (week 1) anti-class II indirect-pathway CD4 T cell response (Figure 2C). In contrast, adoptive transfer of donor DCs into C57BL/6 recipients of a bm12.Kd.IE heart allograft at a late time point (day 28) after transplantation partially restored the late TEa CD4 T cell response (Figure 2D). Parallel experiments, in which DCs from recipients of bm12.kd.IE heart grafts were stained with “YAe” clonotypic antibody, confirmed that expression of I-E peptide epitope for TEa CD4 T cell recognition mirrored the presence and survival of the donor hematopoietic fraction (Figure 2E). As expected, *recipient* hematopoietic cells were also required for TEa CD4 T cell responses, reflecting their role as professional APCs for I-A^b^-restricted allopeptide presentation (Figure 2C). Additional experiments incorporating T- and B-cell-deficient *Rag2*^*−/−*^ mice as recipients of bm12.kd.IE heart allografts surprisingly revealed that the rapid destruction of donor hematopoietic cells by C57BL/6 recipients was not a consequence of innate NK cell recognition but was instead mediated by C57BL/6 adaptive alloresponses (Figure S3).

**Figure 3:**
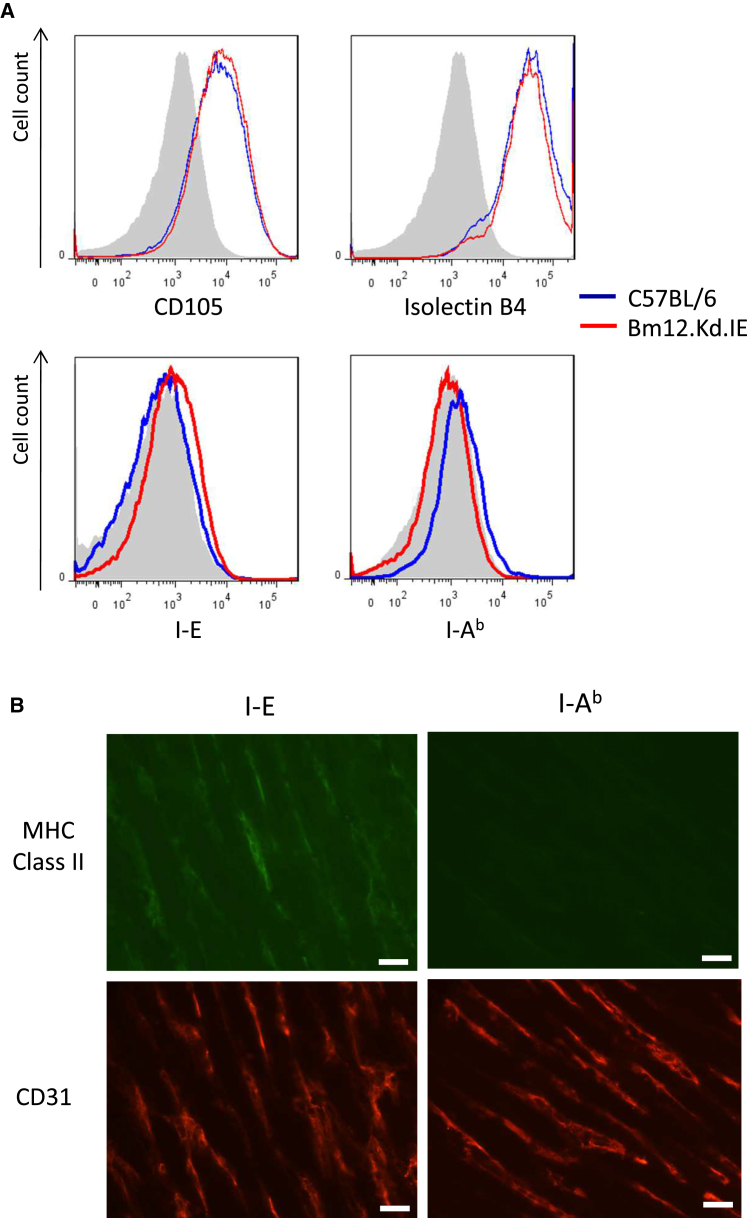
Endothelial Expression of MHC Class II Antigen (A) C57BL/6 (blue) and bm12.kd.IE (red) endothelial cells were cultured from neonatal cardiomyocytes, with representative flow cytometric overlay histograms confirming (top panels) upregulated expression of CD105 and isolectin B4. (B) Cultured C57BL/6 endothelial cells expressed exclusively MHC class II I-Ab, whereas cultured bm12.kd.IE endothelial cells expressed exclusively MHC class II I-E antigens (lower panels: shaded histograms isotype control antibody). Immunofluorescent staining with anti-CD31 (endothelial) antibody and with either anti-I-E or anti-I-Ab antibodies of cryostat sections of bm12.Kd.IE heart allografts explanted 1 week after transplantation into C57BL/6 recipients is shown (scale bars, 20 μm). Data are representative of one experiment (A) and three independent experiments (B), with n = 5 mice per group.

Although MHC class II is generally not thought to be expressed by resting murine endothelium, activation-induced upregulation, as typically occurs within the endothelium of rejecting allografts, has been described (Hasegawa et al., 1998, Milton and Fabre, 1985). In support, I-E MHC class II was detectable on cultured bm12.Kd.IE endothelial cells and was evident on immunohistochemical staining of bm12.Kd.IE heart allografts explanted at 1 week (Figure 3).

The rapid decline of the anti-MHC class II indirect-pathway CD4 T cell response is therefore surprising but may reflect transient expression of MHC class II on graft parenchymal cells; memory TEa CD4 T cells (Figure S4), which would be expected to respond to target epitope even when encountered in the absence of costimulatory ligands, proliferated more robustly than naive cells when transferred into C57BL/6 recipients of bm12.Kd.IE heart allografts at the time of transplant but failed to proliferate when transferred at late time points (Figure 2D).

### Persistent Indirect-Pathway CD4 T Cell Activation from Graft Parenchymal Expression of MHC Class I

**Figure 4:**
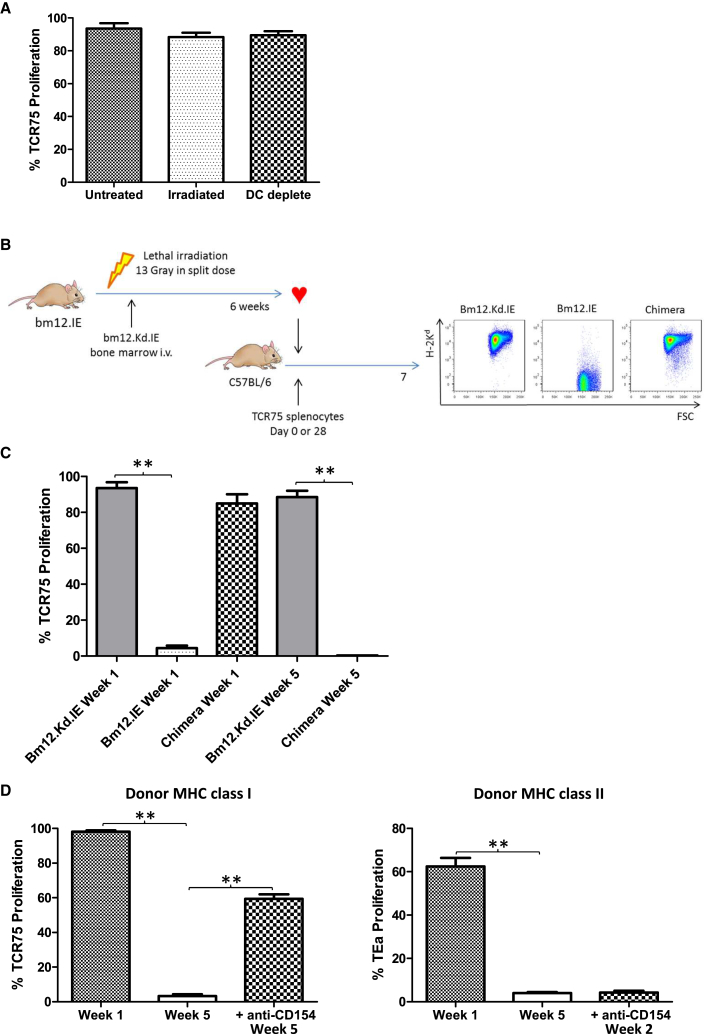
Persistent Indirect-Pathway CD4 T Cell Activation from Graft Parenchymal Expression of MHC Class I (A) Indirect-pathway CD4 T cell responses against MHC class I alloantigen were assessed, as detailed in Figure 2, by quantifying division of CFSE-labeled TCR75 T cells, adoptively transferred into C57BL/6 recipients at transplantation with a bm12.Kd.IE allograft; neither donor lethal irradiation nor donor DC depletion inhibited TCR75 CD4 T cell division. (B) Bone marrow chimeric recipients were created as depicted, by transferring bm12.Kd.IE bone marrow into lethally irradiated bm12.IE mice: lower panels, representative flow cytometry plots demonstrating H-2K^d^ expression in chimeric mice on hematopoietic cells (splenic B cells). (C) Heart allografts from bm12.Kd.IE, bm12.IE, or bm12.Kd.IE→bm12.IE bone marrow chimeric mice were transplanted into C57BL/6 recipients and division of TCR75 CD4 T cells (transferred at the time of, or 5 weeks after, transplantation) quantified. (D) BALB/c heart allografts were transplanted into C57BL/6 recipients that were either left untreated or administered anti-CD154 at transplantation and division of TCR75 CD4 T cells (left panel) or TEa CD4 T cells (right), transferred at the times indicated, quantified. ^∗^p < 0.05, ^∗∗^p < 0.01, and ^∗∗∗^p < 0.001 (Mann-Whitney test in A, C, and D). Data are representative of two independent experiments resulting in n = 5 in each group (A, C, and D; mean and SEM of n = 5 mice per group).

Unlike anti-MHC class II indirect-pathway responses, donor DC depletion or donor irradiation made little impact on CD4 T cell allorecognition of H-2K^d^ allopeptide (Figure 4A). To confirm that late anti-MHC class I indirect-pathway CD4T cell responses were due to recognition of MHC class I alloantigen on graft parenchyma, bm12.Kd.IE bone marrow chimeric mice were created in which H-2K^d^ expression was restricted to the hematopoietic lineage, whereas the graft parenchyma expressed the I-A^bm12^ and I-E alloantigens (Figure 4B). Heart grafts from these mice provoked only a transient anti-MHC class I indirect-pathway response (Figure 4C). Similarly, BALB/c heart allografts, which are rejected within 7 days in C57BL/6 recipients and which presumably do not shed alloantigen thereafter, did not elicit indirect-pathway responses against MHC class I at late time points (Figure 4D). Moreover, treatment of C57BL/6 recipients with anti-CD154 mAb at transplant with a BALB/c heart allograft, a protocol that prevents acute graft rejection but nevertheless results in chronic graft damage (Figure S5; Larsen et al., 1996), restored late TCR75 CD4 T cell responses, whereas indirect-pathway responses against MHC class II antigen were still only short lived (Figure 4D).

### Direct-Pathway CD4 T Cell Responses Are Short Lived and Curtailed by Innate and Adaptive Alloimmunity

**Figure 5:**
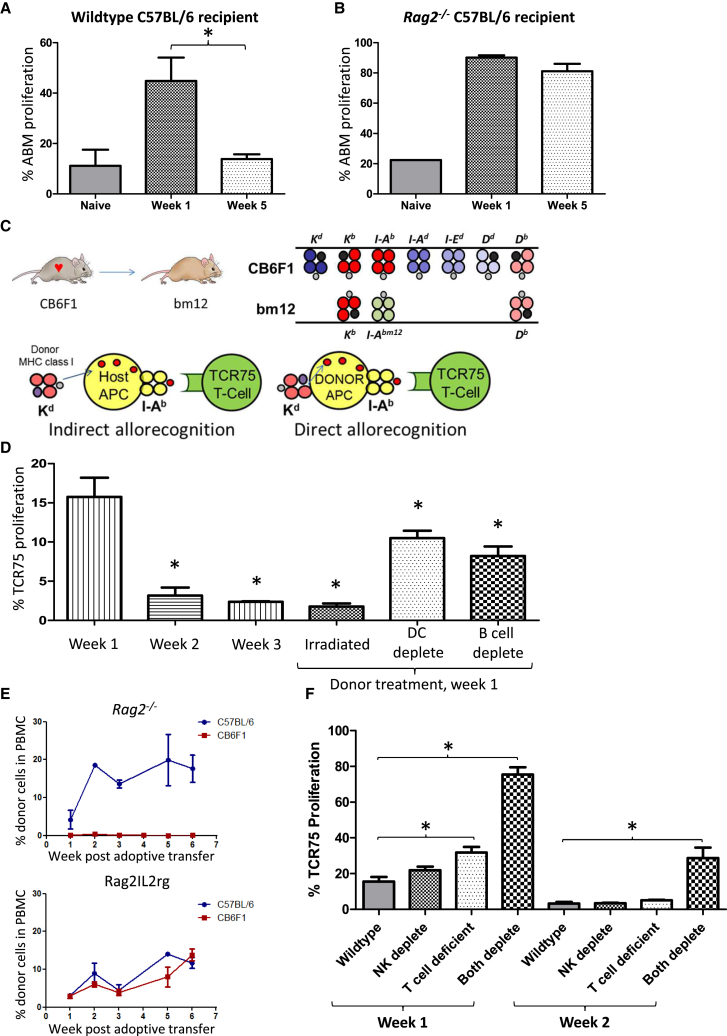
Direct-Pathway CD4 T Cell Responses Are Short Lived and Curtailed by Innate and Adaptive Alloimmunity (A) Direct-pathway CD4 T cell responses were assessed in C57BL/6 recipients of bm12.Kd.IE cardiac allografts by quantifying division of I-A^bm12^-reactive ABM CD4 T cells, adoptively transferred at transplantation or 5 weeks later. Division of ABM CD4 T cells following transfer into naive C57BL/6 recipients is shown for comparison. (B) Direct-pathway responses were prolonged in *Rag2*^*−/−*^ recipients of an unmodified bm12.Kd.IE heart allograft. (C) Direct-pathway CD4 T cell alloresponses were also assessed in bm12 recipients of a BALB/c × C57BL/6 F1 (CB6F1) heart allograft by adoptive transfer of TCR75 CD4 T cells. In this strain combination, TCR75 CD4 T cells do not recognize recipient I-A^bm12^-restricted H-2K^d^ allopeptide but respond to H-2K^d^-peptide presented by I-A^b^ on donor cells. (D) TCR75 CD4 T cell direct-pathway responses are short lived and significantly downregulated by lethal irradiation of the donor or depletion of either donor B cells or DCs. (E) Syngeneic C57BL/6 or allogeneic CB6F1 CD4 T cells were injected into either T- and B-cell-deficient C57BL/6 *Rag2*^*−/−*^ mice (left panel) or Rag2IL2rg mice that additionally lack NK cells (right panel) and the presence of circulating injected cells (expressed as percentage of PBMCs) assessed by flow cytometry. (F) Direct-pathway responses were assessed as in (B) by quantifying division of TCR75 CD4 T cells that were adoptively transferred at the time of, or 1 week after (week 2), transplantation of CB6F1 heart grafts into wild-type, NK cell-depleted, or T-cell-deficient bm12 recipients. ^∗^p < 0.05, ^∗∗^p < 0.01, and ^∗∗∗^p < 0.001 (Mann-Whitney test in A, B, D, and F). Data are representative of two independent experiments resulting in n = 5 in each group (A, B, D, and F; mean and SEM of n = 5 mice per group) and one experiment with n = 3 in each group (E).

The longevity of direct-pathway CD4 T cell allorecognition was assessed in C57BL/6 recipients of bm12.Kd.IE heart grafts by adoptive transfer of I-A^bm12^ reactive ABM CD4 T cells. As for anti-MHC class II indirect-pathway CD4 T cells, direct allorecognition was short lived (Figure 5A) and prolonged upon transplantation of bm12.Kd.IE heart grafts into C57BL/6 *Rag2*^*−/−*^ recipients (Figure 5B). It was notable, however, that ABM CD4 T cells proliferated weakly upon transfer into naive C57BL/6 hosts (Figure 5A), suggesting a degree of cross-reactivity to H-2^b^ antigens. To avoid this, and to control for concerns that the different responses observed with the various populations of transgenic T cells reflected differences in affinity of TCR for target epitope, heart allografts from CB6F1 donors (BALB/c × C57BL/6 F1) were transplanted into bm12 recipients and direct-pathway responses assessed by transfer of TCR75 CD4 T cells. TCR75 CD4 T cells do not recognize I-A^bm12^ restricted antigen (Figure S1; Conlon et al., 2012b); hence, in this model, I-A^b^ / H-2K^d^-peptide target epitope is only expressed on donor APCs and TCR75 T cell division acts as marker for exclusively direct-pathway responses (Figure 5C). In accord with the ABM CD4 T cell transfer studies, TCR75 CD4 T cell division was only detectable in the first week after transplantation and abrogated in recipients of irradiated hearts (Figure 5D). Similarly, depletion of either donor B cells or DCs prior to heart graft retrieval significantly reduced the direct-pathway TCR75 CD4 T cell response (Figure 5D).

In contrast to the bm12.Kd.IE to C57BL/6 model, donor CB6F1 cells are cleared rapidly by host (C57BL/6) NK cell recognition (Figure 5E). However, prolongation of direct-pathway TCR75 CD4 T cell recognition was only achieved in recipients lacking both NK cells and CD4 T cells (Figure 5F), and the response at week 2 was still substantially weaker than that observed at week 1, presumably reflecting the expected half-life of DCs is at most a few weeks (Merad et al., 2007, Merad and Manz, 2009). Hence, the duration of direct-pathway activation is normally limited by both innate and adaptive alloimmune recognition of donor APCs, and even when both are incapacitated, the short lifespan of the donor APC fraction rapidly terminates the response.

### Late Allopeptide Presentation Shapes the Dynamics of the Endogenous CD4 T Cell Alloresponse

**Figure 6:**
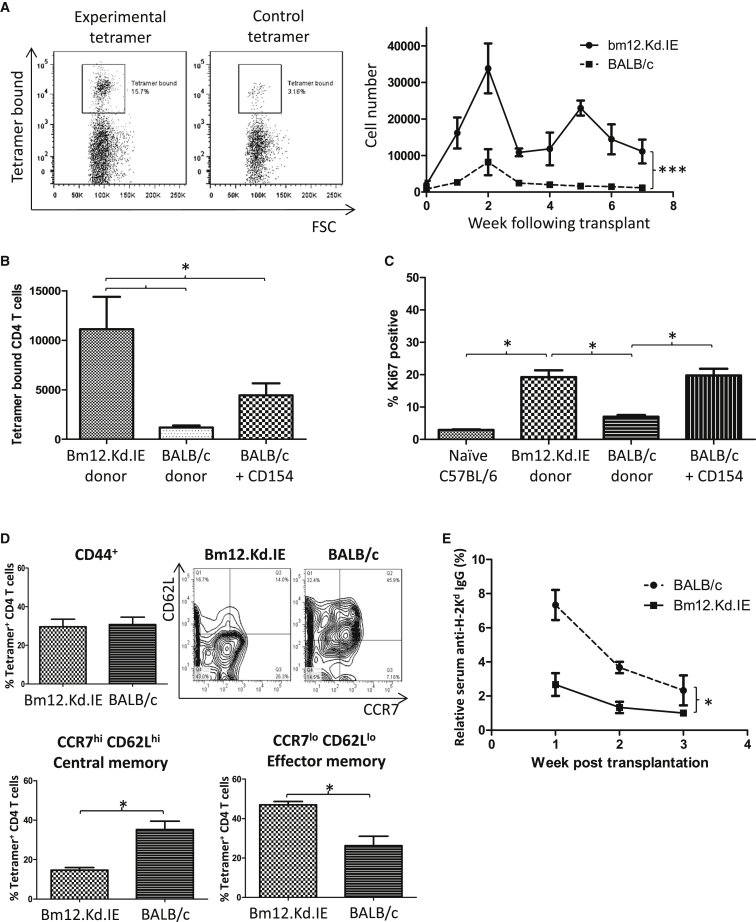
Late Allopeptide Presentation Shapes the Dynamics of the Endogenous CD4 T Cell Alloresponse (A) The endogenous H-2K^d^_54-68_ allopeptide-specific splenic CD4 T cell subset in C57BL/6 recipients of bm12.Kd.IE or BALB/c heart allografts was identified by labeling with H-2K^d^_54–68_/I-A^b^ tetramers: representative flow cytometry plots of binding of splenocytes purified from bm12.Kd.IE heart grafted recipients to (left panels) H-2K^d^_54–68_/I-A^b^ tetramer (experimental) or to control I-A^b^ tetramer bound with human CLIP_87–101_ (class-II-associated invariant chain peptide) and quantification of absolute numbers of tetramer-positive cells present in C57BL/6 recipients at weekly intervals after transplant of bm12.Kd.IE or BALB/c heart allografts (right panel). (B) Prevention of acute rejection by anti-CD154 mAb treatment of C57BL/6 recipients of a BALB/c heart allograft augments numbers of H-2K^d^-allopeptide-specific CD4 T cells 7 weeks after transplant. (C) H-2K^d^-allopeptide-specific CD4 T cells in cell cycle, identified by Ki-67 labeling 5 weeks after transplantation and expressed as percentage of total tetramer-positive cells. (D) Within the antigen-experienced CD44^hi^ tetramer-positive CD4 T cell population (top left panel), CCR7^hi^CD62L^hi^ central (bottom left) and CCR7^lo^CD62L^lo^ effector (bottom right) memory T cells were quantified (gating strategy shown on representative flow cytometry plot) 7 weeks after transplantation of BALB/c or bm12.Kd.IE heart allografts into C57BL/6 recipients. (E) Anti H-2K^d^ IgG alloantibody responses to a B6.K^d^ heart allograft in *Tcrbd*^*−/−*^ recipients, treated with anti-CD154 mAb and reconstituted with splenic CD4 T cells purified from C57BL/6 recipients 5 weeks after transplantation with BALB/c or bm12.Kd.IE heart allografts. ^∗^p < 0.05, ^∗∗^p < 0.01, and ^∗∗∗^p < 0.001 (two-way ANOVA in A and E and Mann-Whitney test in B–D). Data are representative of one experiment with n = 4 in each group (A–C, and E; mean and SEM of n = 4 mice per group) and of two independent experiments with a total of n = 5 in each group (D).

The confirmation, from the above adoptive transfer experiments of TCR75 CD4 T cells, that MHC class I allopeptide epitope is expressed at late time points in recipients of chronically rejecting heart allografts raises the question how this late presentation influences dynamics of the response of the endogenous alloreactive CD4 T cell population. To address this, H-2K^d^-peptide-specific host T cell responses were mapped by labeling splenocytes from transplanted mice with K^d^_54-68_ / I-A^b^ tetramers using a similar approach to that described for CD4 T cell responses against conventional protein antigen (Tubo et al., 2013). Following transplantation with a bm12.Kd.IE cardiac allograft, H-2K^d^ allopeptide-specific CD4 T cells underwent typical expansion, contraction, and memory phases (Figure 6A), but in comparison to challenge with a BALB/c heart allograft (Figure 6A), the expansion phase was much more marked and approximately 10,000-fold more cells persisted into the memory phase. Similar levels of H-2K^d^ alloantigen are expressed on BALB/c and bm12.Kd.IE cardiomyocytes (Figure S6); hence, the increased number of H-2K^d^-specific CD4 T cells at late time points following challenge with a bm12.Kd.IE heart allograft is presumably due to ongoing allopeptide presentation. In support, a less-profound contraction in the allospecific CD4 T cell population occurred in C57BL/6 recipients of a BALB/c heart allograft and that received anti-CD154 antibody at transplantation (Figure 6B), and this late expansion in recipients of chronically rejecting heart allografts correlated with continued division of the H-2K^d^ allopeptide-specific CD4 T cell population (Figure 6C).

Antigen persistence is associated with CD4 T cell exhaustion and defective anamnestic responses (Crawford et al., 2014). Surface phenotyping of the K^d^_54-68_/I-A^b^ tetramer^pos^ CD4 T cell population revealed that the percentage of antigen-experienced CD44^hi^ CD4 T cells was comparable 5 weeks after transplantation with a BALB/c or bm12.Kd.IE heart allograft but that the antigen-experienced CD4 T cells in recipients of a bm12.Kd.IE heart graft were predominantly CD44^hi^CCR7^lo^CD62L^lo^ effector memory, whereas those in BALB/c heart-grafted recipients were more skewed toward CD44^hi^CCR7^hi^CD62L^hi^ central memory (Figure 6D). To assess the functional consequences of this difference, T-cell-deficient *Tcrbd*^*−/−*^ C57BL/6 mice were reconstituted with CD4 T cells purified from recipients of bm12.Kd.IE or BALB/c heart grafts and the reconstituted *Tcrbd*^*−/−*^ mice then challenged with a C57BL/6 donor heart that expressed H-2K^d^ as a transgene (B6.K^d^; Honjo et al., 2004) while simultaneously receiving anti-CD154 mAb. We reasoned that only established H-2K^d^-specific memory CD4 T cells would be able to provide co-stimulation-independent help for development of anti-H-2K^d^ alloantibody responses against the B6.K^d^ graft (Conlon et al., 2012a). Whereas anti-H-2K^d^ alloantibody was not observed in *Tcrbd*^*−/−*^ recipients reconstituted with CD4 T cells from bm12.Kd-IE grafted mice, weak but consistent anti-H-2K^d^ alloantibody responses developed in *Tcrbd*^*−/−*^ recipients reconstituted with CD4 T cells from BALB/c-grafted mice (Figure 6E), despite the vastly reduced number of H-2K^d^ allopeptide-specific CD4 T cells present in this latter group at late time points after transplantation (Figure 6B). Thus, chronic allopeptide presentation drives continual division of the responding alloreactive CD4 T cell population, with persistence of a greatly expanded population but with functional evidence of altered memory development.

## Discussion

Transplantation is unique in that MHC alloantigen can be recognized by host T cells by at least two distinct pathways. By modifying the standard technique of transfer of CFSE-labeled TCR-transgenic CD4 T cells, our experiments confirm that direct-pathway CD4 T cell allorecognition is short lived and limited by innate and/or adaptive alloimmune-mediated elimination of donor APCs. We also detail that indirect-pathway responses to chronically rejecting allografts are heterogeneous and vary according to target alloantigen, with responses against MHC class II peptides also limited by rapid destruction of donor hematopoietic cells.

Fundamental to our experimental approach was the design of a transplant model wherein direct and indirect T cell alloresponses could be assessed simultaneously and in which proliferation of a particular alloreactive T cell population when transferred at transplantation acted as control for the same population transferred at later time points. Thus, interpretation of results should not be confounded by potential differences in TCR affinity between the various TCR-transgenic clones; such concerns are further obviated by the development of additional models that enabled H-2K^d^-allopeptide-specific TCR75 CD4 T cells to act either exclusively via the indirect pathway or exclusively via the direct pathway. Additionally, to reconcile observations obtained from adoptive transfer of TCR-transgenic CD4 T cells with events occurring within the endogenous alloreactive CD4 T cell population, we used synthetic MHC class II I-A^b^/allopeptide tetrameric complexes to assess host CD4 T cell responses against the same dominant H-2K^d^ epitope as recognized by TCR75 CD4 T cells. This approach has been detailed recently for responses against conventional protein antigen (Tubo et al., 2013), but our studies revealed that the alloreactive CD4 T cell responses in the chronic and acute rejection models differ profoundly. In chronic rejection, less-profound contraction of the alloreactive CD4 T cell population occurred, with a markedly augmented maintenance phase. This presumably reflects continual cell division from failure to clear target allopeptide epitope, but whether this was due to ongoing cell cycling of the original alloreactive population or to late recruitment of naive alloreactive CD4 T cells (Lin et al., 2010) was not assessed.

Our findings raise the question whether the continued division and expansion of class I allopeptide-specific CD4 T cells contributes to progression of allograft vasculopathy. Our experiments were not designed specifically to address this, and it is plausible that chronically stimulated CD4 T cells are unable to mediate allograft rejection, as suggested by our data demonstrating that some aspects of allospecific CD4 T cell memory are inhibited by the continual presence of target epitope. Against this, preservation of limited anti-viral function has been described for exhausted CD4 T cells (Yi et al., 2009), and similarly, chronic salmonella infection is controlled by late CD4 T cell responses (Nelson et al., 2013). Notwithstanding, an intriguing possibility raised by our findings is that in “tolerant” recipients, the class I allopeptide-specific CD4 T cell population will be either static or dividing only minimally. Although the technical challenges are daunting, one could envisage that, in the future, tetramer characterization of the division profile of the responding alloreactive CD4 T cell population may be used clinically to identify patients at risk of chronic rejection or those in whom immunosuppression can be safely discontinued.

What then are the implications of our findings for clinical transplantation? It has been suggested that expression of MHC class II is different within human organ allografts, in that human endothelial cells upregulate MHC class II in response to IFN-γ, albeit levels are still lower than for MHC class I (Piotti et al., 2014, Rose, 1998). However, it is also clear, as we demonstrated in our model, that murine endothelium can express MHC class II, and although this expression is unable to activate direct-pathway CD4 T cell responses (Kreisel et al., 2004), it is essential for those responses to then autonomously effect graft rejection (Grazia et al., 2004). Hence, available evidence suggests that the parenchymal components of murine and human organ allografts express MHC class II. Cultured human endothelial cells can activate CD4 T cell in vitro (Adams et al., 1992, Hughes et al., 1990), although this has not been a consistent finding (Ma and Pober, 1998). Whether the parenchymal expression of MHC class II alloantigen on human allografts can drive direct-pathway CD4 T cell activation has not been tested and seems unlikely, because clinical studies report this response to be short lived (Baker et al., 2001). This further suggests that, despite concurrent administration of immunosuppression, donor hematopoietic cells are rapidly eliminated in human recipients, and it is notable that the T cell response against MHC class II alloantigen was similarly brief in our BALB/c to C57BL/6 heart transplant model, in which acute rejection was avoided by concurrent administration of anti-CD154 antibody.

Irrespective of inter-species discrepancies in immune structure and function, our principal finding that murine indirect-pathway CD4 T cell responses vary considerably according to target alloantigen is likely to be relevant to human transplantation, albeit there may be differences in the alloantigen targeted or in the constancy of expression of its epitope. Understanding the relative timing and contribution of direct and indirect CD4 T cell alloresponses to allograft rejection has become all the more important following the recent introduction of alloantigen-specific regulatory T cell (T-reg cell) therapy to clinical transplantation (Brunstein et al., 2011, Di Ianni et al., 2011, Newell et al., 2011, Trzonkowski et al., 2009, Wood et al., 2012). Our results suggest that, for a particular recipient, the success of alloantigen-specific T-reg cell therapy will be governed by whether target epitope is expressed concurrently and provide an explanation for why, in certain animal models, T-reg cells with indirect allospecificity are more effective than those with direct allospecificity in preventing chronic rejection (Joffre et al., 2008, Tsang et al., 2008). T-reg cells with direct allospecificity are likely to be effective only if injected in the immediate post-transplant period. Indirect-pathway T-reg cells hold greater potential for administration at later time points after transplantation as a means of controlling development of chronic rejection, but the success of such an approach will require consideration of which indirect-pathway CD4 T cell responses are active.

## Experimental Procedures

### Animals

Wild-type (WT) C57BL/6 (B6; H-2^b^) and BALB/c (H-2^d^) and CB6F1 (C57BL/6 × Balb/c F1 – H-2^bd^) mice were purchased from Charles River Laboratories. Bm12 mice - B6(C)-H2-Ab1bm12/KhEgJ (H-2^bm12^), B6.CD11c-DTR (H-2^b^ - B6.FVB-Tg), BALB/c.CD11c-DTR (H-2^d^ - C.FVB-Tg), and *Tcrbd*^−/−^ B6 (H-2^b^) mice (Jung et al., 2002) were purchased from The Jackson Laboratory. BALB/c.CD11c-DTR mice were crossed with B6 to obtain CB6F1 (H-2^bd^) mice expressing the DTR gene (CB6F1.CD11c-DTR). *Tcrbd*^−/−^ B6 mice were crossed with bm12 mice to create bm12-*Tcrbd*^−/−^ T-cell-deficient mice. C57BL/6 *Rag2*^*−/−*^ mice (H-2^b^) were gifted by Prof. T. Rabbitts (University of Cambridge). Rag2IL2rg (Song et al., 2010; H-2^b^ *Rag2*^*−/−*^ lacking the IL-2 receptor γ chain; NK cell deficient) was kindly gifted by Dr. Francesco Colucci (University of Cambridge). C57BL/6 mice that lack expression of I-A^b^ but express surface I-E antigen on APCs due to I-Eα gene incorporation (ABOIE) were gifted by Prof. C. Benoist (Joslin Diabetes Center; Le Meur et al., 1985). C57BL/6-Tg(K^d^)RPb mice (B6.Kd; Honjo et al., 2000) expressing H-2K^d^ were gifted by Dr. R.P. Bucy (University of Alabama). Bm12.Kd.IE mice were created in house by first crossing bm12 × B6.Kd, selecting from the offspring those expressing I-A^bm12^ and H-2K^d^, but not I-A^b^ (bm12.Kd), and then crossing them with ABOIE mice and selecting offspring expressing I-A^bm12^, I-E, and H-2K^d^, but not I-A^b^. For TCR transgenic animals, *Rag2*^*−/−*^ TEa mice (H-2^b^; TEa), specific for I-A^b^-restricted I-E^d^_52-68_ peptide, were gifted by Prof. A. Rudensky (University of Washington; Grubin et al., 1997); *Rag1*^*−/−*^ TCR75 mice (H-2^b^; TCR75), specific for I-A^b^-restricted H-2K^d^_54-68_ peptide, were gifted by Prof. P. Bucy (University of Alabama; Honjo et al., 2004); *Rag2*^*−/−*^ ABM mice (H-2^b^; ABM), I-A^bm12^ restricted, were gifted by Dr. T. Crompton (Imperial College; Bäckström et al., 1998); and *Rag1*^*−/−*^ Marilyn mice (H-2^b^; Mar), specific for the I-A^b^-restricted H-Y *dby*_608–622_ peptide, were gifted by Dr. Di Scott (Imperial College; Lantz et al., 2000). Mice were bred and maintained in specific-pathogen-free animal facilities, and all experiments were approved by the UK Home Office under the Animals (Scientific Procedures) Act 1986.

### Generation of Bone Marrow Chimeras

To create chimeric mice that were CD11c-DTR^+^ transgenic only in bone marrow cells, C57BL/6 mice were lethally irradiated (2 × 6.5 Gy) and reconstituted with 2 × 10^7^ bone marrow cells from C57BL/6.CD11c-DTR mice. The DCs in these mice remained susceptible to diphtheria toxin (DT) treatment, but the mice were significantly more resistant to overall toxicity (Sapoznikov and Jung, 2008). Bm12.Kd.IE mice with expression of class I H-2K^d^ restricted to the hematopoietic compartment were generated by lethal irradiation of bm12.IE mice and reconstituted with 2 × 10^7^ bone marrow cells from bm12.Kd.IE mice. Chimerism was confirmed by flow cytometric analysis of PBMCs at least 4 weeks following reconstitution.

### Skin and Heterotopic Cardiac Transplantation

Full-thickness tail skin was sutured as 1 cm^2^ grafts onto the recipient’s back. Vascularized cardiac allografts were transplanted intra-abdominally using the technique of Corry and colleagues (Corry et al., 1973). Heart graft rejection was defined as cessation of palpable myocardial contraction, confirmed at explant for histology. In certain experiments, recipients of BALB/c heart allografts were injected i.p. with 500 μg anti-CD154 mAb (clone MR-1; BE0017-1; Bio X Cell) on days −2, 0, 2, and 4 in relation to transplantation, a protocol that prevents acute allograft rejection but that results in development of chronic allograft vasculopathy.

### In Vivo Depletion and Transfer of Donor and Recipient Leukocyte Subsets

Hematopoietic cells were depleted as previously described (I.G.H., J.M.A., S.J.F. Harper, E. Wlodek, M.C.N., M.S.Q., R.M., K.S.-P., E.M.B., J.A.B., M.R. Clatworthy, T.M.C., and G.J.P., unpublished data) by lethal irradiation (2 × 6.5 Gy) of donor mice 7 days before heart allograft procurement. B6.CD11c-DTR, BALB/c.CD11c-DTR, and CB6F1.CD11c-DTR donors and C57BL/6 (BL/6.CD11cDTR) chimeric heart graft recipients were treated with i.p. 32 ng/g DT (List Biological Laboratories) on days −5, −3, and −1 (donors) or thrice weekly in recipients. To deplete CD4 T cells, mice were treated with 1 mg i.p. anti-CD4-depleting mAb (rat IgG2b; clone YTS 191.1; hybridoma from European Collection of Cell Cultures; Health Protection Agency; Porton Down U.) on days −5, −3, and +1 relative to transplantation and weekly thereafter. To deplete B cells, mice were treated with 250 μg i.p. depleting anti-CD20 mAb (18B12; IgG2a gifted by Cherie Butts, Biogen Idec) on day −7 and, for recipients, fortnightly thereafter. To deplete NK cells, mice were treated with 0.5 mg i.p. depleting anti-NK1.1 mAb (mouse 1gG2a; clone PK13 – hybridoma; ATCC-LGC Standards Partnership) on days −2, 0, and +2 and weekly thereafter. Cell depletion was confirmed by flow cytometry.

In certain experiments, *Rag2*^*−/−*^ and/or Rag2IL2rg mice were adoptively transferred i.v. 1 × 10^7^ donor CD4 T cells (purified with anti-mouse CD4 MicroBeads; Miltenyi Biotec) or 1 × 10^7^ donor B cells (purified with anti-mouse CD19 MicroBeads) using an autoMACS Separator (Miltenyi Biotec).

In another experiment, 1 × 10^7^ memory CD4 T cells were purified from recipients of bm12.Kd.IE or BALB/c heart grafts 6 weeks after transplant using magnetic bead separation as above and transferred into naive C57BL/6 *Tcrbd*^*−/−*^ mice that were subsequently challenged with B6.Kd cardiac allografts.

### Quantification of Circulating Antibodies

Serum samples were collected from experimental mice at intervals and analyzed for the presence of anti–H-2K^d^ IgG alloantibody by ELISA as previously described (Conlon et al., 2012b). In anti-I-E alloantibody quantified by flow cytometry, briefly, serum was serially diluted and incubated with ABOIE bone-marrow-derived DCs (BMDCs) (prepared as previously described; Sivaganesh et al., 2013); bound alloantibody was detected with FITC-conjugated anti-mouse IgG mAb (STAR 70; Serotec) and analyzed. The geometric mean-channel fluorescence was plotted against dilution, and the AUC was calculated as for H-2K^d^ alloantibody.

Circulating autoantibody levels were determined by HEp-2 indirect immunofluorescence as previously described (Callaghan et al., 2012).

### Mouse Heart Homogenization and Endothelial Cell Culture

Mouse hearts were finely diced, digested with collagenase (1 mg/ml collagenase A [Roche], 1 mg/ml DNase1 [Roche], and 2% FCS [Sigma-Aldrich] in DMEM) and passed through a 40-μm nylon strainer to yield a single-cell suspension.

For endothelial cell culture, 10- to 14-day neonatal hearts were homogenized as above and endothelial cells isolated by incubating with biotin-conjugated antibodies against CD31 (clone MEC 13.3; BD PharMingen), CD105 (clone MJ7/18; BioLegend), and isolectin B4 (clone B-1205; Vector) followed by anti-biotin MicroBeads and purified using an autoMACS Separator (both Miltenyi Biotec). Endothelial cells were cultured overnight in growth medium (HEPES-buffered DMEM with 10% FCS, 100 IU/ml penicillin-streptomycin, and 2 mM L-glutamine; Life Technologies) in tissue culture flasks (Nunc; Thermo Scientific) pre-coated with 1% gelatin (Sigma-Aldrich) in PBS. Non-adherent cells were removed and endothelial cell growth factor (E9640; Sigma-Aldrich) added to the culture medium. Cells were passaged by incubation with trypsin-EDTA solution (Life Technologies) at 37°C until cells detached.

### Histopathology and Immunohistology

Splenic GCs were identified by double-labeling 7 μm cryostat sections with rat anti-mouse B220 (clone RA3-6B2; BD PharMingen) detected with Cy3-conjugated goat anti-rat IgG (clone 112-165-143; Jackson ImmunoResearch Laboratories) and biotinylated rat anti-mouse GL-7 (clone Gl-7; E-Bioscience) detected with Cy2-streptavidin (Jackson ImmunoResearch Laboratories).

Endothelial MHC class II expression was detected in donor heart 7 μm cryostat sections by double labeling with rat anti-mouse CD31 (clone MEC 13.3; BD Pharmingen) detected with Cy3-conjugated goat anti-rat IgG and FITC-conjugated rat anti-mouse I-E (clone 14-4-45; BD Pharmingen) followed by FITC-conjugated rat anti-mouse I-A^b^ (clone AF6-120.11; BD PharMingen). Sections were counterstained with 20% Harris’ hematoxylin (Sigma-Aldrich).

Cardiac allograft vasculopathy was assessed on elastin van Gieson-stained paraffin sections by morphometric analysis as previously described (Callaghan et al., 2012). All elastin-positive vessels in each section were evaluated, with approximately ten vessels/heart analyzed.

### Flow Cytometry

APC-CD4 (clone GK1.5), PE-Cy7-CD4 (clone GK1.5), PE-CD8 (clone 53-6.7), PercP-CD8 (clone 53-6.7), APC-Cy7-CD11b (clone MI/70), PE-CD11c (clone HL3), FITC-CD11c (clone HL3), PE-CD19 (clone 1D3), APC-CD19 (clone 1D3), PercP-CD25 (clone PC61), APC-CD44 (clone IM7), APC-CD62L (clone MEL-14), FITC-CD69 (clone H1.2F3), biotin-CD105 (clone MJ7/18), biotin-isolectin B4 (clone B-1205), FITC-I-E (clone 14-4-45), FITC-I-A^b^ (clone AF6-120.11), biotin-NK1.1 (clone PK136), APC-Cy7-CD90.1/Thy1.1 (clone OX-7), biotin-Vα2 (clone B20.1), PE-Vβ6 (clone RR4-7), PE-Vβ8 (clone OX-7), PE-Vβ8.3 (clone 1B3.3), biotin-YA-e (clone YA-e), and PE-K^d^ (clone SF1-1) were purchased from BD PharMingen. Peripheral blood (erythrocyte-depleted by 0.17 M NH4Cl lysis) and splenic single-cell suspensions were blocked with anti-mouse CD16/CD32 (clone 2.4G2) before staining with the relevant Abs and dead cell exclusion dye 7-aminoactinomycin (both from BD PharMingen). Biotinylated Abs were detected by allophycocyanin-conjugated streptavidin (Invitrogen), and cells were analyzed on a FACSCanto II flow cytometer with FACSDiva software (Becton Dickinson).

For subsequent intracellular antigen detection, surface-labeled cells were fixed and permeabilized using the Cytofix/Cytoperm kit according to manufacturer’s instructions (BD Biosciences) and incubated with antibodies to Ki67 (clone B56; BD PharMingen).

For tetramer labeling, spleen single-cell suspensions in FACS buffer were blocked with anti-CD16/CD32 and incubated with PE-conjugated MHC class II I-A^b^ tetramers presenting MHC class I K^d^ peptide 54-68 (QEGPEYWEEQTQRAK), kindly gifted by the NIH Core Tetramer facility. After 1 hr at 37°C, cells were washed and tetramer-bound cells were enriched using anti-PE microbeads (Moon et al., 2007, Tubo et al., 2013; Miltenyi Biotech) and an Automacs separator. Additional cell surface antigens were then labeled for flow cytometry as above, and antigen-specific CD4 T cells were identified as tetramer- and CD4-positive but negative for CD19, CD11c, CD11b, and CD8. Antigen-specific CD4 T cells were enumerated after MACS separation by Trucount analysis according to manufacturer’s instructions (BD Biosciences).

### CFSE CD4 T Cell Proliferation

Proliferation of splenocytes from TCR75, TEa, ABM, and Mar mice were determined by CFSE-labeled cell division at day 7 following adoptive transfer as previously described (Conlon et al., 2012b) using PE-Cy7-conjugated anti-CD4 plus APC-Cy7-conjugated anti-CD90.1/Thy 1.1 (congenic marker) to identify TCR75, PE-conjugated anti-Vβ8 to identify ABM, biotin-conjugated Vα2 and PE-conjugated anti-Vβ6 to identify Tea, and PE-conjugated anti-Vβ8.3 to identify Mar cells. CFSE-labeled splenocytes were transferred either early, at transplantation, or late on day 28 after transplant (to measure CD4 T cell alloresponses during the first and fifth week, respectively). In one experiment, proliferation of TEa splenocytes was examined late after transfer of 5 × 106 donor bm12.Kd.IE BMDCs (generated as described above). Proliferation was quantified using FlowJo (Treestar) as the percentage of cells from the original parent population that had undergone division.

### Statistical Analysis

Data were presented as mean ± SEM where appropriate. Mann-Whitney U test was used for analysis of nonparametric data. Two-way ANOVA was employed for comparison of anti-H-2K^d^, anti-I-E, and anti-nuclear antibody levels. Graft survival was depicted using Kaplan-Meier analysis and groups compared by log rank (Mantel-Cox) testing. Analysis was conducted using GraphPad 4 (Graph-Pad Software). Values of p < 0.05 were considered significant.

## Author Contributions

Conceptualization, J.M.A., J.A.B., and G.J.P.; Investigation, J.M.A., M.C.N., T.M.C., M.S.Q., I.G.H., R.M., and K.S.-P.; Designed, Developed, and Produced Essential Reagents: R.W. and M.C.N.; Writing – Original Draft, J.M.A. and G.J.P.; Writing – Review & Editing, J.M.A., M.C.N., T.M.C., M.S.Q., I.G.H., R.M., R.W., K.S.-P., E.M.B., and J.A.B.

## Supplemental Information

Document S1. Figures S1–S6

Document S2. Article plus Supplemental Information
