# Supplementary material for: Diversity of the CD4 T Cell Alloresponse: The Short and the Long of It
Source: Cell Rep. 2016 Jan 21;14(5):1232–45. doi: 10.1016/j.celrep.2015.12.099 (PMC5405053; doi:10.1016/j.celrep.2015.12.099)
Supplement: Document S1. Figures S1–S6 [file mmc1.pdf]

Cell Reports, Volume *14*

Supplemental Information

Diversity of the CD4 T Cell Alloresponse:

The Short and the Long of It

Jason M. Ali, Margaret C. Negus, Thomas M. Conlon, Ines G. Harper, M. Saeed Qureshi, Reza Motallebzadeh, Richard Willis, Kourosh Saeb-Parsy, Eleanor M. Bolton, J. Andrew Bradley, and Gavin J. Pettigrew

Supplemental Figures

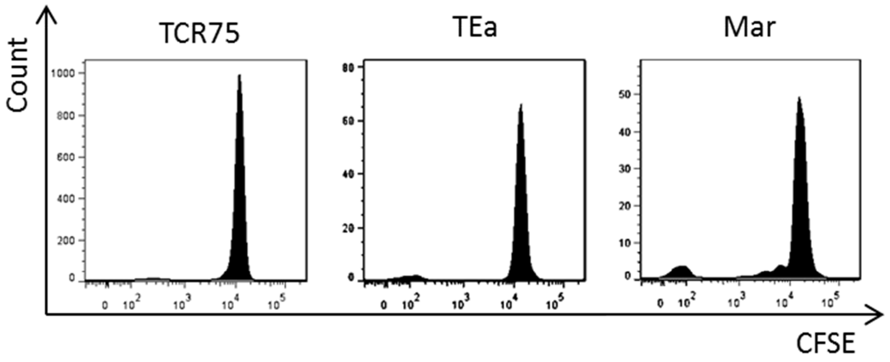

Figure S1: Alloreactive TCR-transgenic CD4 T cell lines do not recognise target alloantigen when restricted for I-A^{bm12}, related to figure 1.

Male BALB/c heart allografts were transplanted into female bm12 recipients and division of CFSE-labelled TCR75, TEa and Marilyn (Mar) CD4 T cells, transferred at transplantation, assessed one week later by flow cytometric identification of the transferred cells within the splenic CD4 T cell population. Representative flow cytometry plots.

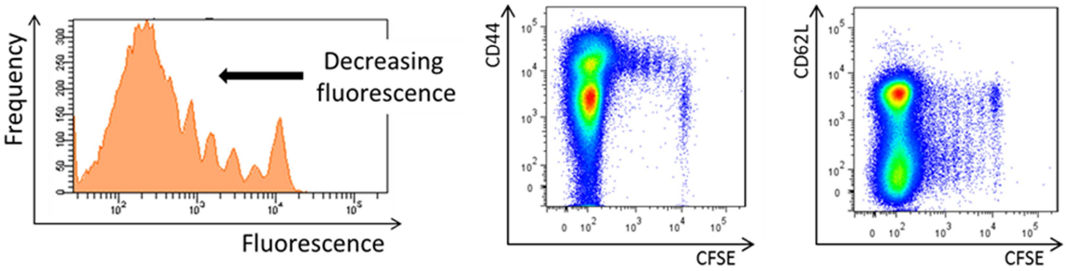

Figure S2: Representative flow cytometry histogram of CFSE division profiles, related to figure 2.

CFSE-labelled TEa CD4 T cells were transferred into C57BL/6 recipients at transplantation with a bm12.Kd.IE heart allograft. Division was assessed six days later by flow cytometric identification of the transferred cells within the splenic CD4 T cell population. Flow cytometry plots demonstrate increased expression of CD44 and decreased expression of CD62L as the transferred cells divide.

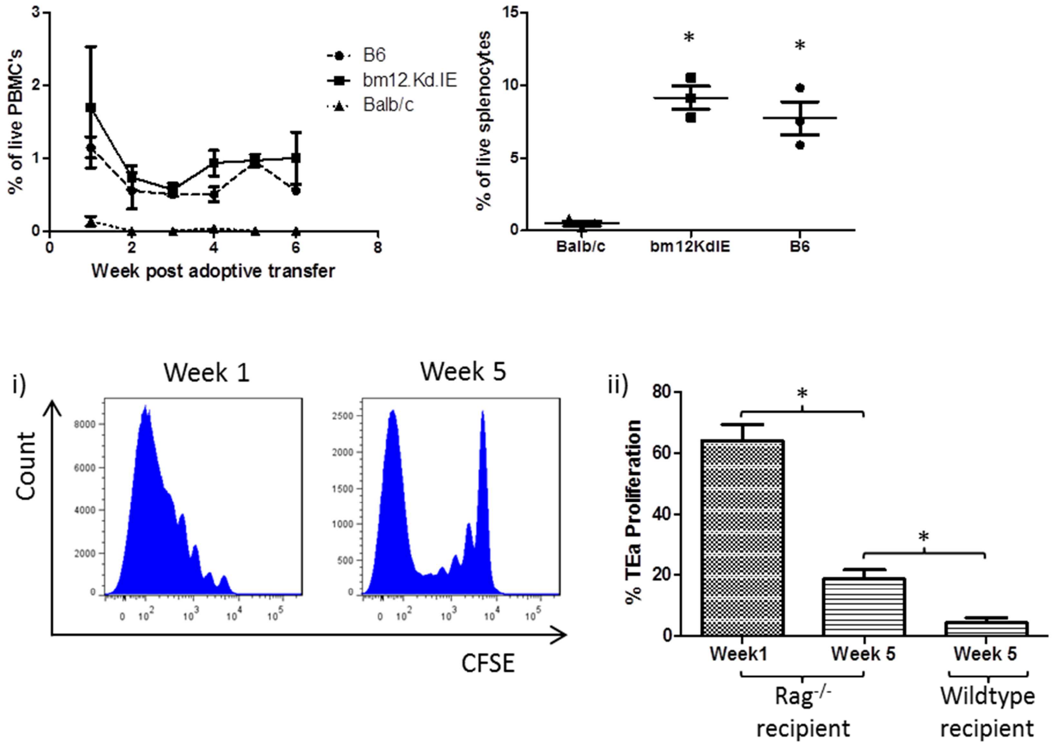

Figure S3: Passenger bm12.Kd.IE lymphocytes avoid innate NK allorecognition, but are killed rapidly by adaptive alloimmunity in C57BL/6 recipients, related to figure 2.

B lymphocytes were purified from C57BL/6 (B6), bm12.Kd.IE or BALB/c mice by magnetic bead separation and transferred into *Rag2*$^{-/-}$ C57BL/6 mice. **(a)** Mice were bled weekly and injected cells, identified by flow cytometry, expressed as a percentage of the peripheral blood mononuclear cell (PBMC) fraction. **(b)** Mice were culled at week 6 and injected cells, identified in the spleen by flow cytometry, expressed as a percentage of the live splenocyte fraction. **(c)** CFSE-labelled TEa CD4 T cells were transferred into *Rag2*$^{-/-}$ C57BL/6 recipients of bm12.Kd.IE heart allografts either at transplantation or 28 days after transplant (representative flow cytometric histograms depicted in left panel), and division of the transferred cells quantified as described in the legend to figure 2 (right panel). Included for comparison is the proliferation of TEa CD4 T cells when transferred into wild-type C57BL/6 recipients 28 days after transplantation of bm12.Kd.IE heart grafts (as depicted in Figure 2). *$P < 0.05$. TEa CD4 T cell division in *Rag2*$^{-/-}$ recipients at week 5 is significantly greater than that observed in wild-type recipients at the same time point, but significantly less than that observed in *Rag2*$^{-/-}$ recipients at week 1, suggesting that adaptive immune-mediated killing of donor antigen presenting cells (APCs) rapidly curtails indirect-pathway CD4 T cell alloresponses against MHC class II allopeptide, but that in the absence of immune recognition, donor APC are a naturally short-lived population, surviving for only a few weeks.

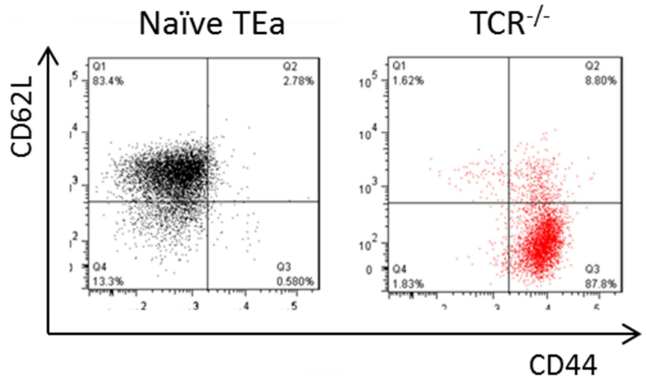

Figure S4: Generation of TEa memory CD4 T cells, related to figure 2.

C57BL/6 *Tcrbd*$^{-/-}$ mice were reconstituted with splenocytes from *Rag2*$^{-/-}$ TEa mice and challenged with ABOIE skin grafts that express an isolated I-E mismatch. Representative flow cytometry plots comparing memory formation (CD44^{hi}CD62L^{lo}) in the splenic CD4 T cell population in naïve *Rag2*$^{-/-}$ TEa mice and in the reconstituted mice six weeks after challenge. Memory CD4 T cells were purified by magnetic bead separation from reconstituted mice 6 weeks after challenge, CFSE-labelled and transferred into C57BL/6 recipients of bm12.Kd.IE heart allografts as detailed in Figure 2.

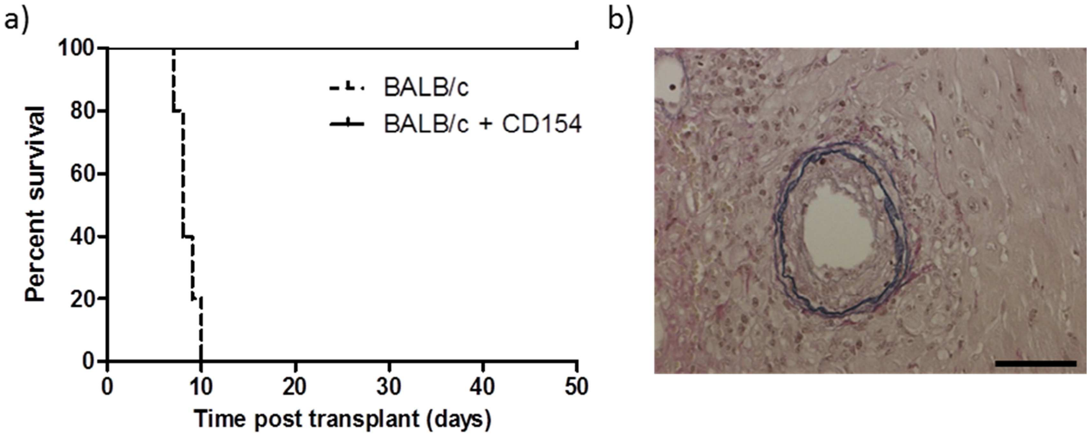

Figure S5: Development of chronic allograft vasculopathy in C57BL/6 recipients of BALB/c heart allografts, related to figure 3.

Treatment of C57BL/6 recipients of BALB/c heart allografts with anti-CD154 mAb at transplant prevents acute rejection and results in long-term graft survival; **(a)** Kaplan-Meier survival plots; but heart grafts develop progressive allograft vasculopathy; **(b)** representative photomicrographs of elastin van Gieson stained paraffin sections depicting typical fibroproliferative arterial intimal thickening observed in rejecting heart allografts (Scale bar, 100 μm).

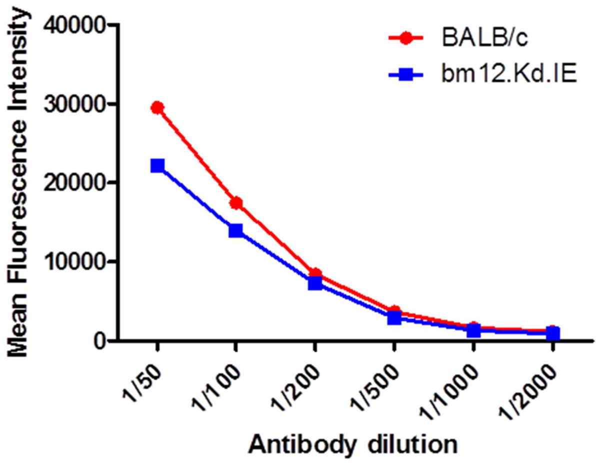

Figure S6: Expression of H-2K^d MHC class I antigen in bm12.Kd.IE and BALB/c hearts, related to figure 5.

BALB/c and bm12.Kd.IE hearts were digested with collagenase and expression of H-2K^d antigen assessed by flow cytometry following incubation of aliquots of single cell homogenates with serial dilutions of anti-H-2K^d antibody.
